# Intensive surveillance, rapid response and border collaboration for malaria elimination: China Yunnan’s ‘‘3 + 1’’strategy

**DOI:** 10.1186/s12936-021-03931-8

**Published:** 2021-10-09

**Authors:** Jian-Wei Xu, Zu-Rui Lin, Yao-Wu Zhou, Rogan Lee, Hai-Mo Shen, Xiao-Dong Sun, Qi-Yan Chen, Kai-Xia Duan, Peng Tian, Chun-Li Ding, Shi-Yan Xu, Hui Liu, Hong-Ning Zhou

**Affiliations:** 1 Yunnan Institute of Parasitic Diseases, Yunnan Provincial Centre of Malaria Research, Yunnan Provincial Key Laboratory of Vector-Borne Diseases Control and Research, Yunnan Institute of Parasitic Diseases Innovative Team of Key Techniques for Vector Borne Disease Control and Prevention (Developing), Training Base of International Scientific Exchange and Education in Tropical Diseases for South and Southeast Asia, Puer, 665000 China; 2 The Centre for Infectious Diseases and Microbiology, New South Wales Health Pathology and Westmead Clinical School, The University of Sydney, Westmead Hospital, Sydney, NSW 214 Australia; 3 Chinese Center for Disease Control and Prevention, National Institute of Parasitic Diseases, Shanghai, 200025 China

**Keywords:** Border-spill malaria, 3 + 1 strategy, Intensive surveillance, Rapid response, Elimination, Collaboration, China

## Abstract

**Background:**

Eliminating malaria and preventing re-establishment of malaria transmission in border areas requires universal coverage of malaria surveillance and a rapid response to any threats (i.e. malaria cues) of re-establishing transmission.

**Main text:**

Strategy 1: Intensive interventions within 2.5 km-wide perimeter along the border to prevent border-spill malaria. The area within 2.5 km along the international border is the travel radius of anopheline mosquitoes. Comprehensive interventions should include: (1) proactive and passive case detection, (2) intensive vector surveillance, (3) evidence-based vector control, and (4) evidence-based preventative treatment with anti-malarial drugs. Strategy 2: Community-based malaria detection and screening of migrants and travellers in frontier townships. Un-permitted travellers cross borders frequently and present in frontier townships. Maintenance of intensified malaria surveillance should include: (1) passive malaria detection in the township hospitals, (2) seek assistance from villager leaders and health workers to monitor cross border travellers, and refer febrile patients to the township hospitals and (3) the county’s Centre for Disease Control and Prevention maintain regular proactive case detection. Strategy 3: Universal coverage of malaria surveillance to detect malaria cues. Passive detection should be consolidated into the normal health service. Health services personnel should remain vigilant to ensure universal coverage of malaria detection and react promptly to any malaria cues. Strategy + 1: Strong collaborative support with neighbouring countries. Based on the agreement between the two countries, integrated control strategies should be carried out to reduce malaria burden for both countries. There should be a clear focus on the border areas between neighbouring countries.

**Conclusion:**

The 3 + 1 strategy is an experience summary of border malaria control and elimination, and then contributed to malaria elimination in Yunnan’s border areas, China. Nevertheless, Yunnan still has remaining challenges of re-establishment of malaria transmission in the border areas, and the 3 + 1 strategy should still be carried out.

## Background

The goal of the World Health Organization (WHO) is to achieve at least 90% global reduction of mortality rate and case incidence and to eliminate malaria from at least 35 endemic countries by 2030 compared with 2015 [1]. This goal also aims to eliminate malaria in all countries in the Greater Mekong Subregion (GMS) [2]. However, malaria still remains high on the list of diseases that causes major health burdens, globally. According to the WHO World Malaria Reports 2019 and 2020, progress has slowed or even stalled. The rate of reduction of malaria mortality slowed over the period 2016–2018 compared to the period 2010–2015. An estimated 228 million cases of malaria were reported worldwide in 2018 [3] and 229 million malaria cases in 2019 in 87 malaria endemic countries [4]. In China, malaria transmission has been interrupted with zero indigenous cases reported since 2017 [6, 7]. As a notable feat for a country that reported 30 million cases of the disease annually in the 1940s following a 70-year effort, China has eliminated malaria. The WHO has awarded China a malaria-free certification on 30 June 2021[8]. However, cross border malaria transmission from the GMS is a significant impediment to malaria elimination in the GMS [5]. China is now shifting malaria control strategies from elimination to preventing re-establishment of transmission [7, 9], so the importation of malaria from endemic countries especially from its southern borders needs to be halted.

Fourteen other countries share borders with China, these include Korea, Mongolia, Russia, Kazakhstan, Kyrgyzstan, Tajikistan, Afghanistan, Pakistan, India, Nepal, Bhutan, Myanmar, Laos and Vietnam. Yunnan, in southwestern China, is the province that faces the highest threat of re-establishment of malaria transmission along its border. This unique province has a total population of 48 million, and there is a population of 8 783 788 (2020) in its 25 border counties that shares 4,060 kms of border with Myanmar, Laos and Vietnam. Border counties are defined as the border areas in China. In Yunnan Province, about 70% of the border population consists of farmers who mostly live in rural areas, and about 30% of the border population is governmental staff, traders and service sector staff who mostly live in towns. In addition, Yunnan has similar malaria ecology and vector species with five other countries in the GMS [10]. In order to reduce imported malaria for the purpose of preventing re-establishment of transmission, the 3 + 1 strategy was developed to intensify malaria surveillance, to respond rapidly to imported malaria, and to work in collaboration with neighbouring countries. Here the combination of the 3 + 1 strategy and followed up with the 1–3-7 rapid response [11] employed only to Yunnan and not to the country as a whole for border malaria elimination, and the remaining challenges in preventing re-establishment of transmission are presented.

### Strategy 1: Intensive malaria interventions within 2.5 km wide perimeter along the border to prevent spread into China

#### Challenges faced

Along the border areas, imported malaria is a continual threat to maintaining the elimination status in China. In particular, malaria parasites that spills over from endemic countries that share a common border with China have interfered with the progress of malaria elimination [12]. Border-spill malaria, described in this article, is a type of imported malaria carried by infected anophelines which fly over the boundary from endemic areas of neighboring countries to reintroduce malaria along China’s border areas. The border-spill malaria is associated with the distance travelled by female anopheline mosquitoes. The results of 291 anopheline mosquito mark-release-recapture (MMRR) experiments in 143 localities around the world estimated that the mean distance travelled (MDT) of female anophelines was not more than 2.5 km [13]. Based on this study, a strategy of comprehensive and intensive interventions was developed to prevent border-spill malaria along a 2.5 km wide perimeter on the international border with China.

#### Interventions

This package includes proactive and passive detections for the malaria pathogen, intensified vector surveillance and prompt action based on the surveillance results. When any malaria cases are detected, a rapid response would follow China’s ‘‘1-3-7’’ strategy. This involves reporting of malaria cases within one day, confirmation and investigation of malaria cases within three days, and an appropriate public health response to prevent further transmission within seven days [11]. Malaria surveillance within 2.5 km perimeter along the common boundary of China and the neighbouring country is used to estimate the endemicity of malaria along the border. Where China’s border area is at risk of border-spill malaria, long-lasting insecticidal bed nets (LLINs) are normally provided to local residents at the rate of two persons per net. Meanwhile, intensified vector surveillance should be carried out by light traps, collecting mosquitoes off indoor walls and ceilings, capturing mosquitoes indoors off human subjects and this should be extend to capturing off humans subjects outdoors during malaria transmitting season (April to October) each year. The number of malaria cases and vector surveillance data are analysed to determine whether there is a need for indoor residual spraying (IRS) with insecticides. IRS should be conducted when *Anopheles minimus* (local principal vector) is found and there are increasing malaria cases diagnosed in local health facilities both in China and the neighboring country. Preventive treatment with anti-malarial drugs should be delivered to local residents [14] when the IRS is unable to control increasing border-spill malaria cases within China. Similar interventions should be conducted to decrease malaria incidence along the border with agreement and collaboration of neighbouring countries to prevent border-spill malaria into China.

### Strategy 2: Community-based malaria surveillance and response at frontier townships

#### Challenges faced

The Chinese government administrative system is divided into six levels, the first is national followed by provincial to prefecture to county to township and finally to village level. Individuals who cross borders into China without permits frequently present in townships or towns along the international boundary (frontier townships). The median population size for a frontier township is around 15,000 in Yunnan. There are no natural or artificial barriers on the boundary and migration control is only available at the formal ports of entry and exit. People of the same ethnic groups live on either side of the border and some of them are relatives or close friends. Local residents in these areas walk freely across the border. Travellers who do not have entry permits into China include illegal migrant workers, traders, visitors of relatives and friends. They are at high risk of carrying malaria infections and can easily reintroduce malaria or even re-establish malaria transmission in China’s border areas [15, 16]. Intensified malaria surveillance should, therefore, be maintained in these frontier townships. The strategy of community-based surveillance and timely targeted response should be used to prevent re-establishment of transmission.

#### Interventions

this should involve township hospitals who are responsible for the passive detection. In addition, village leaders and village health workers should actively monitor migrants and refer febrile patients, especially foreign visitors from the endemic areas, to the township hospitals for malaria testing. The county centre for disease control and prevention (CDC) and the township hospital regularly conduct proactive case detection to screen migrants from the endemic areas. Remote villages (including villages under strategy 1 and 3) are deemed to be those which are either more than 5 km walking distance or more than 30 min motor vehicle ride to the nearest township hospital. These remote villages do have trained health or malaria workers who use rapid diagnostic tests (RDT) to detect febrile patients for malaria. The village health workers should report any positive cases identified by RDT to the county CDC and then the county CDC will rapidly follow up with microscopic investigation as evidence-base interventions following the ‘‘1-3-7’’ rapid response strategy [11, 17].

### Strategy 3: Universal coverage of malaria surveillance to detect malaria cues

**Fig. 1:**
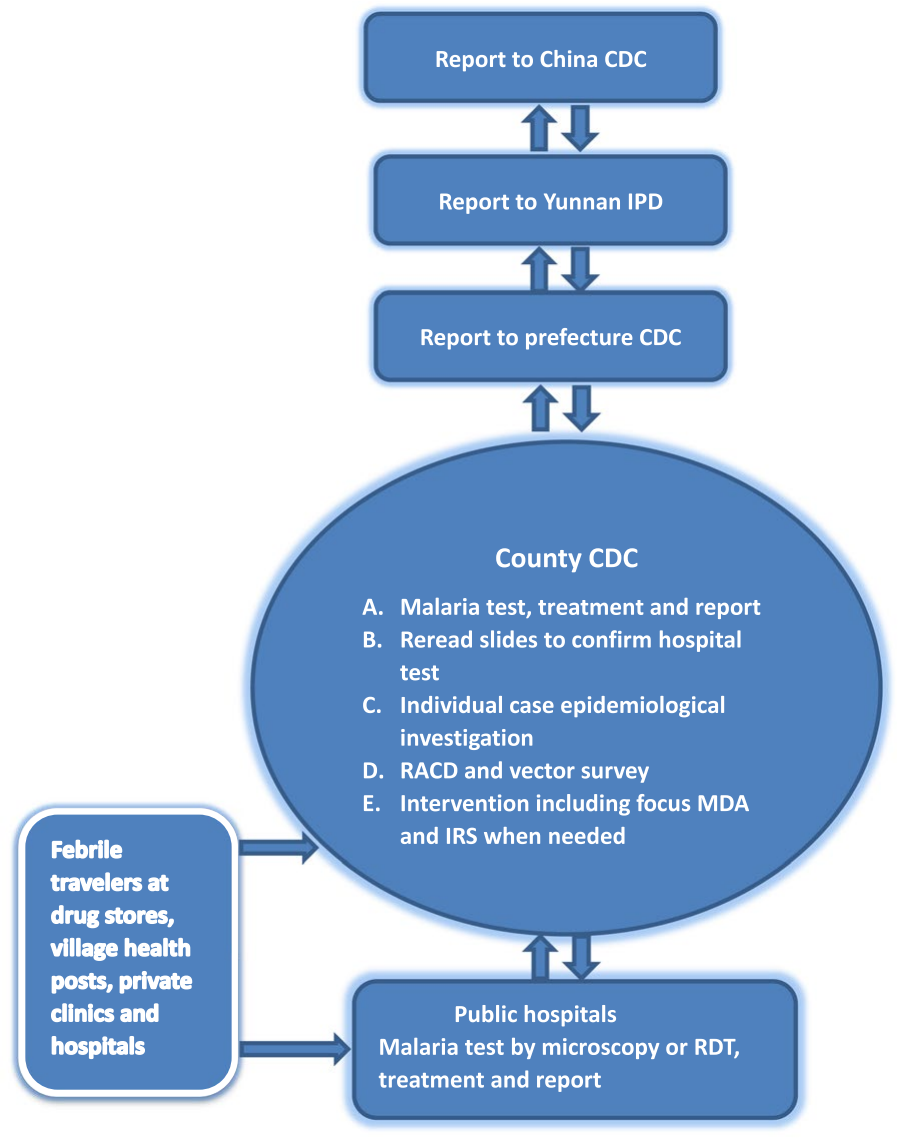
A reporting flow chart of malaria surveillance and response in Yunnan. CDC = Center for Disease Control and Prevention; IPD = Institute of Parasitic Diseases; RACD = reactive case detection; MDA = mass drug administration; IRS = indoor residual spraying with insecticides; RDT = rapid diagnosis test

A county is a unit of malaria elimination in China. The median population size of a county is about 350,000 in Yunnan. To ensure that universal coverage of malaria detection occurs, passive detection of malaria cases should be consolidated into the normal health service, continually maintain vigilance of health personnel, and any suspected malaria cues should be promptly addressed [12]. In this article, a malaria cue is either a malaria infection involving a positive malaria test by microscopy or RDTs or febrile patients who are negative by tests at the normal health service, but have stayed overnight in endemic countries. These febrile patients need further investigation with a more sensitive polymerase chain reaction (PCR) test or an expert microscopist at a reference laboratory for malaria. Hospital-based malaria case detection provides mandatory feed back to the county CDC within 24 h and a centralized response to malaria cues is activated to prevent re-establishment of transmission across Yunnan Province (Fig. 1) [11, 17]. There are a total of 129 counties in Yunnan. All county and township hospitals and selected private hospitals in the 25 border counties should conduct laboratory test for malaria surveillance. These designated hospitals perform malaria diagnosis by microscopy or RDTs and report their results into the web-based China Information System for Disease Control and Prevention (CISDCP) in real time. Other health facilities, including village, private clinics and hospitals, and also drug stores, are required to refer clinically suspected malaria cases to the designated hospitals (Fig. 1). Meanwhile, annual training or retraining courses are used to maintain competency of hospital staff and stimulate vigilance for malaria diagnosis and treatment of imported malaria. The local health authority coordinates the referral of febrile patients with suspected malaria from the private hospital sectors and drug stores to the designated hospital and the county CDC [17]. The CDC at each level from central government to county should provide information, education and communication (IEC) material to travellers to promote individual protection by use of bed nets and preventive treatment with anti-malarial drugs during their stay in malaria endemic countries. Travellers returning back to China and developing fevers should seek prompt laboratory diagnosis and appropriate treatment from the CDC and designated hospitals after returning from endemic countries. The CDC also provide IEC material to both private hospital sector and drug store personnel to promote the reporting of suspected malaria cases to the CDC and the designated hospitals. The travellers are informed that they can obtain free services for malaria, including free anti-malarial drugs, laboratory testing, mosquito repellents, long-lasting insecticidal mosquito nets (LLINs), consultation and IEC materials. In addition, the social health insurance will cover their diagnosis and treatment for malaria in the designated hospitals. This universal coverage of malaria detection and prompt treatment could reduce malaria spread to other areas in China.

The county CDC acts as the response center for malaria in each county (Fig. 1). When malaria notifications are received from the CISDCP, the county CDC responds by following the ‘‘1-3-7’’ strategy [11]. When local transmission of malaria is detected and positive cases are found among family members or neighbours. Treatment by focused mass drug administration (MDA) with presumed exposure should then be given to the index case and people in the surrounding homes according to the national drug policy and treatment guidelines [14].

### Strategy + 1: Solid collaboration with neighbouring countries

#### Collaboration and interventions

To reduce the risk of re-establishment of transmission in China, a malaria elimination programme needs to have collaboration with countries that export malaria. Neighbouring countries with Yunnan Province should engage in the border elimination process, especially, in the areas where border-spill malaria occurs [18]. A solid collaboration of cross border malaria control should involve current malaria prevalence meetings, training, data sharing and face to face visits. In addition, it requires effective interventions with agreed strategies and activities between the two countries with common borders.

Integrated interventions by both countries should be carried out to either reduce malaria burden or eliminate malaria along the border areas of endemic neighbours. With the information that adult female anophelines have a flight range of not more than 2.5 km diameter [13], neighbouring endemic countries should concentrate efforts towards a 2.5 km wide perimeter covering their common border with China. The extent of collaborative interventions of the neighbouring country depends on the level of endemicity and China’s ability to offer financial support for malaria control measures [19]. If possible, the optimal strategy should aim to eliminate malaria along the border areas of neighbouring countries. In the case of countries with hyperendemicity, the interventions should be more comprehensive and intensive, including effective treatment to clear parasite reservoirs, vector control to reduce transmission and communicate/educate behavioural changes to promote individual protection against malaria. In addition, continual surveillance for pathogens and their vectors in these neighbouring countries are essential in providing support in reducing the malaria burden or eliminate malaria along the border areas with Yunnan Province, China.

#### Challenges faced

Even if the Central Governments agreed that collaboration of cross border malaria control between two countries, implementation of the agreed activities remains very challenging [19]. The complex system of administration may slow down the implementation of the agreed activities, or even make it impossible to carry out. To simplify the process and overcome barriers caused by different administrative systems in cross border malaria control, the Central Government of both countries can decide on an agreed framework and empower corresponding local government agencies along with civil society organizations to carry out activities with mutual benefit.

#### Achievements

the agreement of cross border malaria control was signed between China and Myanmar on 7 June 2005 [18]. With support from the International Collaboration Department of National Health Commission (the former Ministry of Health) of China, Yunnan has carried out “The joint malaria control project along China–Myanmar Border" since 2005 [19]. The project has expanded to the China–Laos-Vietnam border, and it also includes the control of dengue fever since 2010 [19]. The overall incidence of malaria is low in Vietnam with malaria transmission being interrupted in the north of Vietnam [20, 21]. In the northern provinces of Laos, malaria control has also made rapid progress towards localized elimination goals. Only sporadic malaria cases can now be detected due to cross-border collaboration with neighbouring countries [22]. The overall low incidence of malaria in northern Vietnam and Laos supports the progress of malaria elimination in Yunnan along the China-Vietnam-Lao border [23].

**Fig. 2:**
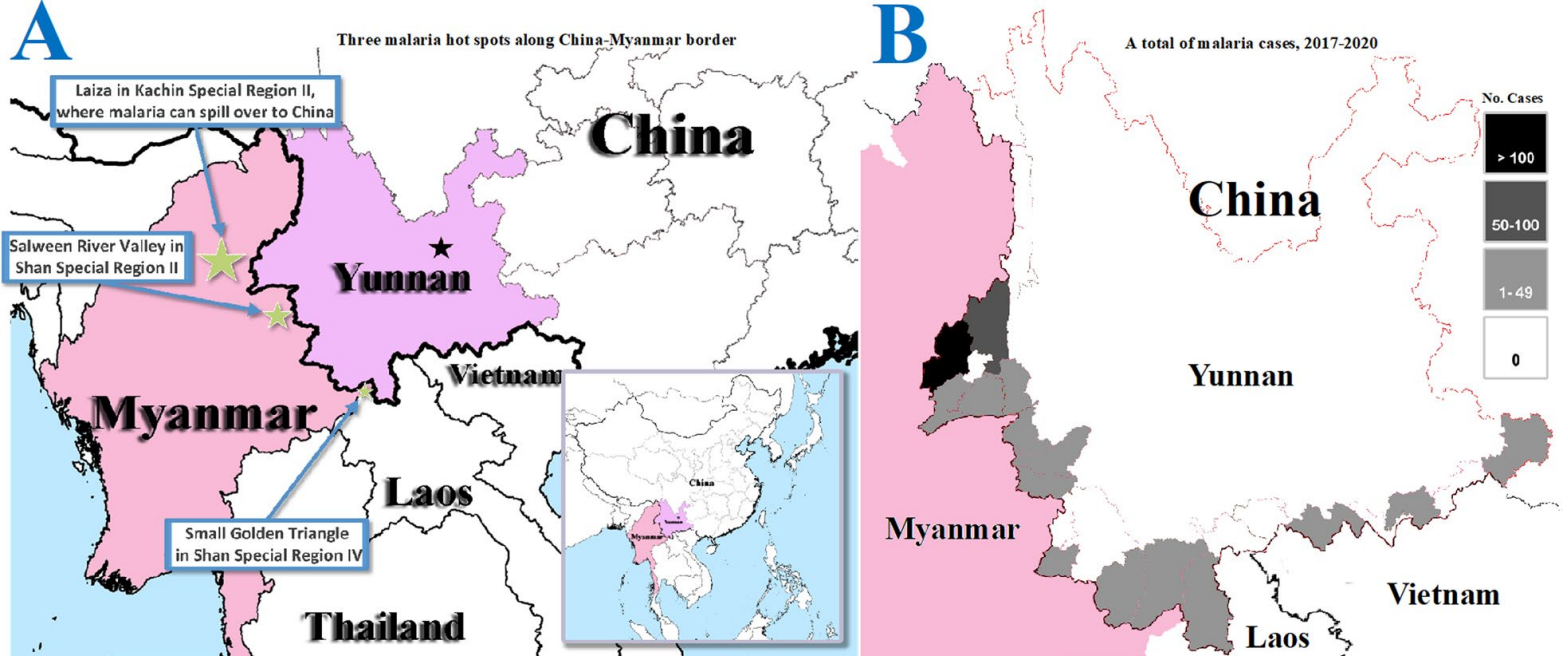
A) Three main malaria hot spots along China-Myanmar border (star size is indicative of the malaria burden and threat to Yunnan Province). B) Risk level in each of 25 border counties based on the total number of imported malaria cases when local transmission was interrupted in China (2017–2020)

The China-Myanmar border is the most difficult area for malaria elimination in Yunnan [24, 25]. The agreement of cross border malaria control between China and Myanmar encourages initiatives between local governmental sectors and non-governmental organizations (NGO) in the both countries [18]. Yunnan Institute of Parasite Diseases (YIPD) and a UK-based NGO running the Health Poverty Action (HPA), have successfully implemented the sixth round of the Global Fund to fight AIDS, Tuberculosis and Malaria (GFATM) project rolled out in July 2007 and the tenth round launched in January 2012. The two GFATM programmes obtained a total budget of US$ 32,512,550. China allocated a total of US$ 17,196,071 (52.9%) to the HPA for control activities in the border areas of Myanmar. When cars, drugs, bed nets and other equipment were included in the budget, more than 70% of the two grants received by China’s CDC were spent in the border areas of Myanmar. The two programmes reduced the malaria burden by 90% in five special regions of Myanmar, decreasing malaria parasite prevalence from 13.6% (95% CI, 12.7–14.6%) in March, 2008, to 1.5% (95% CI, 1.2–2.0%) in November, 2013. More importantly, the two programmes helped the Ethnic Health Organizations (EHO) establishing the primary health care systems in the five special regions of Myanmar. These primary health care systems are now incorporated as the basic health structures for future collaboration in cross border disease control between China and Myanmar [24]. As a result of this strong collaboration, **t**he hyperendemic areas were reduced to only three main hot spots of malaria by 2013, namely, Laiza and nearby areas in Kachin Special Region II (KR2), Salween River Valley in Shan Special Region II (SR2) and Small Golden Triangle in Shan Special Region IV (SR4]) along the China-Myanmar border (Fig. 2). The significant reduction of malaria burden in the border areas of Myanmar has contributed to the success of malaria elimination in China [24, 25].

### Border malaria elimination progress and 3 + 1 strategy development

**Fig. 3:**
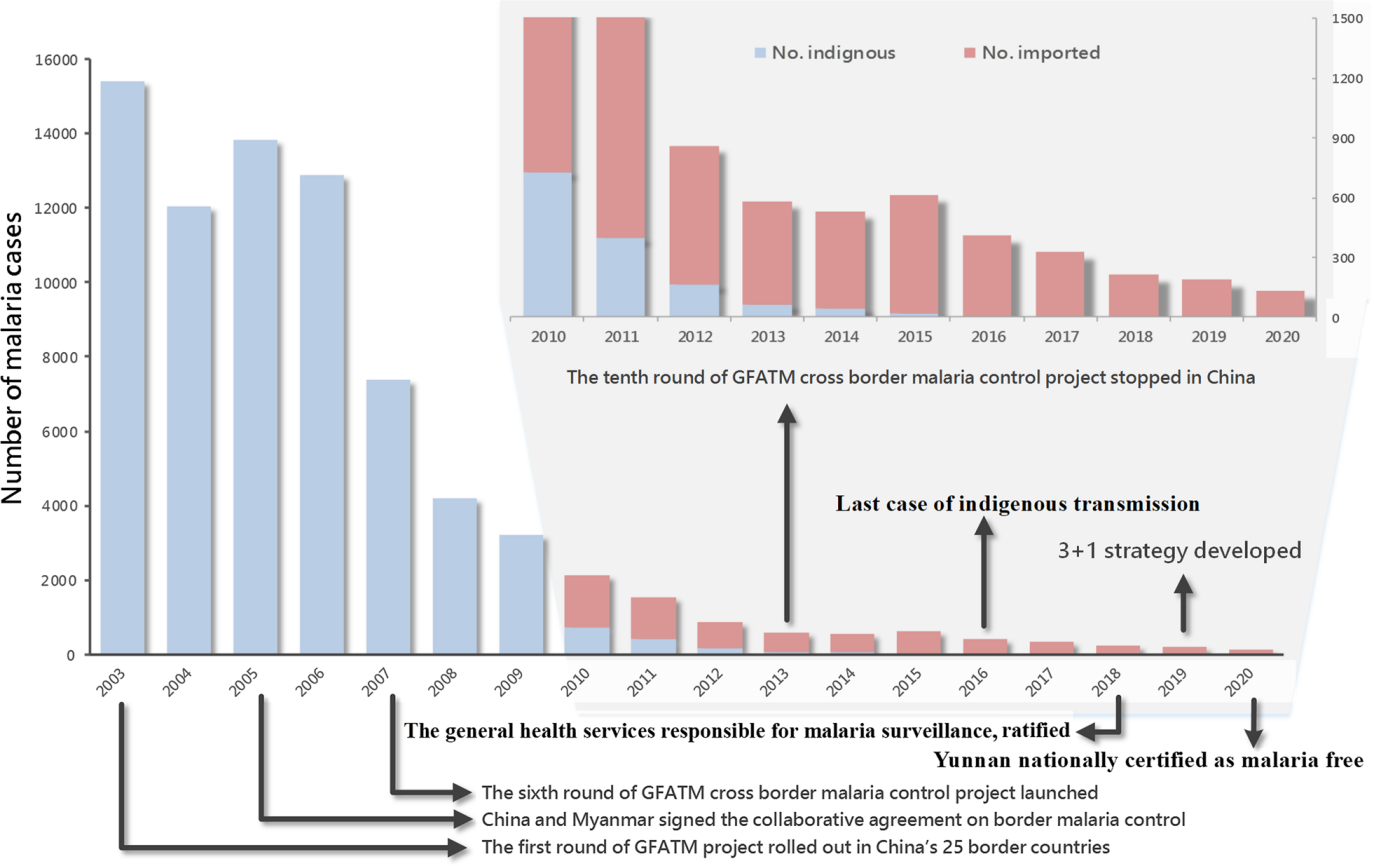
Progress of the malaria elimination programme and application of the 3 + 1 strategy to monitor and control imported malaria into China. GFATM = Global Fund to fight AIDS, Tuberculosis and Malaria

Before the launch of the National Malaria Elimination Programme in July 2010 [26], there was no separation of malaria into indigenous and imported cases in Yunnan province. In January 2003, the first round of the China’s GFATM malaria programme was rolled out in 25 counties along Yunnan’s border. With improvement of both laboratory diagnosis and reporting, a total of 15,431 confirmed malaria cases were reported from Yunnan in 2003 (Fig. 3). When the first collaboration document of cross border malaria control was signed between China and Myanmar in 2005, Yunnan reported a total of 13 845 confirmed malaria cases (9 881 *Plasmodium vivax*, 3 204 *Plasmodium falciparum* and 760 not typed *Plasmodium* spp.). In 19 counties along China-Myanmar border alone, 4,340 (48.9%) of 8,874 malaria cases were categorized as imported malaria from Myanmar in 2006. From July 2007 to December 2013, the sixth and tenth rounds of the China’s GFATM programs were successfully carried out along China-Myanmar border, and then only 518 imported malaria cases occurred in Yunnan in 2013 [24]. China was listed by the World Bank as a mid-high income country in 2012 and was no longer eligible for grants from the GFATM. The second phase of the tenth round of China’s GFATM project was consolidated into the Myanmar’s GFATM project from 2014.

There was a slight resurgence of malaria incidence in Myanmar’s border areas, which led to the increase of imported cases of malaria (593) into Yunnan in 2015 [27, 28]. Yunnan focused on suppressing the resurgence and strengthened its joint action of malaria control in Myanmar’s border areas to minimize the threat. Since then, Yunnan has interrupted the local malaria transmission and remained malaria free from 17 April 2016 when the last case of indigenous malaria was reported [29] (Fig. 3). In March 2018, Yunnan Health and Family Planning Commission (YHFPC) promulgated and ratified the “The Notification on further standardizing malaria elimination work and process”, in which the normal health services were mainly responsible for detecting and reporting malaria cues [17]. In January 2019, the 3 + 1 strategy of malaria elimination is now enforced based on the experiences and the lessons learned in border malaria control and elimination (Fig. 3).

## Discussion and remaining challenges

Border malaria is one of the most intractable problems that countries face today in their path to malaria elimination. This mini review describes the strategy used during the process of malaria from hyperendemicity to elimination along international border in Yunnan Province, China. The achievement is attributable to multifactor, including political commitment, effective interventions, socioeconomic development and changing ecological environment, and the complicated interactions between these factors [23]. Other countries in their path to malaria elimination should develop their own strategy based on their local context, including malaria burden, governing system, health service structure, socioeconomic development and ecology. A malaria programme should reduce annual parasite incidence to less than one per 1 000 person-years prior to orientation from a control to an elimination programme [12]. Border malaria elimination is an intensive intervention, including universal coverage of surveillance to detect malaria cases, rapid response to any malaria cues and effective border collaboration with neighbouring to reduce threats of imported malaria, especially border-spill malaria. Other border areas should do their own intervention trials and a local decision should be made according to their own trial results [30].

Despite China has achieved malaria elimination, there are still three key challenges that affect the implementation of the 3 + 1 strategy to prevent re-establishment of malaria transmission along the international border in Yunnan. First, following the WHO’s recommendation, a malaria alert and response system should replace regular malaria surveillance activities [12]. In China, financing of clinical medicine services is modelled on partial public funding; this also includes the public hospitals. Hospitals charge for cost of medical services. About 60% of patient’s cost is borne by social health insurances and about 40% of the cost is borne by the individual. These costs may influence a febrile patient’s decision as to whether he or she will attend the hospital for malaria diagnosis and treatment. The collaboration of public health, clinical medicine services and social insurance sectors in malaria surveillance needs more strengthening by the county government. Secondly, the threat of re-establishment of transmission remains because malaria can be imported from neighbouring endemic countries. Although the threat from Vietnam and Lao PDR is slight [22, 23], it is still difficult to keep malaria free along China-Myanmar border. An outbreak of *P. falciparum* caused by imported malaria from the Salween River Valley hot spot (Fig. 2) [28, 31] was reported in the Shan Special Region II of Myanmar in June of 2014. Although the outbreak site (a rubber plantation) was about 10 km away from the China-Myanmar border, most of people in the plantation were the Lahu ethnic minority who immigrated into Myanmar from China. China and Myanmar collaboratively controlled the outbreak and prevented malaria exportation to China [28]. Due to the hyperendemicity and military conflict, the KR2 malaria hot spot in Myanmar is a continuous threat to malaria elimination along the border areas of China [32]. From 2017 to 2020, a total of 719 imported malaria cases were reported from the 25 border counties, 678 (94.3%) of them detected in six counties (Yingjiang 452 cases, Tenchong 91, Longchuan 45, Mangshi 41, Ruili 38 and Longling 41) that neighbour with the KR2, and all 46 border-spill malaria cases occurred in Yingjiang County (Fig. 2). An example of border-spill malaria was recently identified in this region. In early November 2019, staff (239) from a Chinese construction team were working on the river banks; this river is the physical border between China and Myanmar. The construction site was 17 kms away from the nearest Chinese community, but there were two camps of internal displaced persons (IDP) on the banks of the Myanmar side of the river. Febrile Chinese workers who returned to their home town for treatment were being reported on 16 November. Once the first case of *P. vivax* malaria was diagnosed at the Mangshi CDC, reactive case detections (RACD) were carried out in the construction site and the worker’s hometowns. The RACD identified a total of 22 *P. vivax* malaria cases, which were considered as border-spill malaria caused by infected anophelines from the IDP camps on Myanmar river banks. A prompt response successfully controlled the malaria outbreak at the construction site to prevent reintroduction into China [33]. This outbreak also tested the efficiency of the malaria surveillance and response system in China [17]. This is an example of how border-spill malaria significantly increases the risk of reintroducing malaria into China (Fig. 2). Finally, Sustained investment of resources should be maintained and the government should not reduce their support once the region becomes malaria free. The technical capacity in malaria diagnosis and treatment may become retrograde when health personnel are under resourced in addition to having a low chance of seeing malaria patients. The same situation for vector control, the technical capacity in anopheline taxonomy and surveillance may also become retrograde. If technical skills and vigilance are not maintained, China’s border areas will be exposed to re-establishment of malaria transmission.

## Conclusion

The 3 + 1 strategy is an experience summary of border malaria control and elimination, which is from practice of border malaria control and elimination, and then contributed to malaria elimination in China’s border areas. Nevertheless, China still has remaining challenges of re-establishment of transmission in the border areas. The 3 + 1 strategy should still be used combined with the 1-3-7 rapid response strategy to prevent re-establishment of malaria transmission in the border areas.

## Data Availability

Not applicable.

## Abbreviations

WHO: World Health Organization
GMS: Greater Mekong Subregion
MMRR: Mosquito mark-release-recapture
MDT: Mean distance travelled
LLINs: Long-lasting insecticidal bed nets
IRS: Indoor residual spraying with insecticides
RDT: Rapid diagnostic tests
RACD: Reactive case detections
CDC: Centre for disease control and prevention
PCR: Polymerase chain reaction
CISDCP: China Information System for Disease Control and Prevention
IEC: Information, education and communication
MDA: Mass drug administration
GFATM: Global Fund to fight AIDS, Tuberculosis and Malaria
IDP: Internal displaced persons
EHO: Ethnic Health Organizations
KR2: Kachin Special Region II: SR2: Shan Special Region II
SR4: Shan Special Region IV
YHFPC: Yunnan Health and Family Planning Commission
